# Calculation of the C_3_A Percentage in High Sulfur Clinker

**DOI:** 10.1155/2010/102146

**Published:** 2010-06-27

**Authors:** Sayed Horkoss, Roger Lteif, Toufic Rizk

**Affiliations:** ^1^Faculty of Sciences, Saint Joseph University, Campus of Sciences and Technologies, Mar Roukos. Mkallès, P.O. Box. 11-514 Riad El Solh, Beirut 11072050, Lebanon; ^2^Plant- Laboratory Department, Cimenterie Nationale S.A.L., Old Tripoli Road, Chekka, North Lebanon. P.O. Box. 11-5101 Riad El Solh, Beirut 11072180, Lebanon

## Abstract

The aim of this paper is to clarify the influence of the clinker SO_3_ on the amount of C_3_A. The calculation of the cement phases percentages is based on the research work, Calculation of the Compounds in Portland Cement, published by Bogue in 1929 .The usage of high sulphur fuels, industrial wastes, and tires changes completely the working condition of Bogue because the assumed phase compositions may change. The results prove that increasing the amount of SO_3_ in the low alkali clinker decreases the percentages of C_3_A due to the high incorporation of alumina in the clinker phases mainly C_2_S and C_3_S. The correlation is linear till the clinker SO_3_ reaches the 2%. Over that the influence of the clinker SO_3_ became undetectable. A new calculation method for the determination of the C_3_A in the high sulphur and low alkali clinker was proposed.

## 1. Introduction

Portland cement is a hydraulic material composed primary of calcium silicates, aluminates, and ferrites. In a rotary kiln, at temperature reaching the 1450°C, clinker nodules are produced from a finely ground, homogenised blend of limestone, shale and iron ore.

The nodules are subsequently ground with gypsum, which serves to control setting, to a fine powder to produce finished Portland cement. The composition and texture (crystal size, abundance, and distribution) of clinker phases result from complex interactions of raw feed chemical and mineralogical composition, particle size distribution, feed homogenization, and the heating and cooling regime.

In order to simplify these phenomena, Bogue [1] proposed an approach for the development of the clinker phases. The ferric oxide (Fe_2_O_3_) reacts with aluminium oxide (Al_2_O_3_) and lime (CaO) to form the tetracalcium aluminoferrite (C_4_AF or Ca_4_Al_2_Fe_2_O_10_). The remaining aluminium oxide reacts with lime to form the tricalcium aluminate (C_3_A or Ca_3_Al_2_O_6_). The lime reacts with the silicate oxide (SiO_2_) to form two calcium silicates phases, the dicalcium silicate (Belite, C_2_S or Ca_2 _SiO_4_) and tricalcium silicate (Alite, C_3_S or Ca_3_SiO_5_). 

Based on the above approach, Bogue proposed four formulae for the calculation of the clinker phase concentrations. 

Increasing the amount of the high sulphur fuels and the level of waste valorisation, in the cement kilns, changes completely the working condition of Bogue. The negative influence of sulphate on the percentages of silicate phases (alite and belite) was earlier detected by the XRD but that on the tricalcium aluminate (C_3_A) is still unclear due to the conclusion contradiction in the reported literature.

## 2. Influence of Sulphur on Silicate Phases

The sulphates reduce the viscosity and surface tension of the clinker liquid phases, shifting the equilibrium of the melt into unstable range which is characterized by low nucleus forming frequency and high growth rate of crystal leading to stabilization of the Belite crystals. The incorporation of sulphur in the Belite stabilizes the Belite structure, whereby the uptake of CaO is inhibited and the formation is suppressed [2]. This phenomenon increases the amount of Belite and decreases that of Alite in the clinker [3]. This reported conclusion was assured by many investigations done later [2, 4, 5].

## 3. Influence of Sulphur on Aluminates C_3_A (Ca_3_Al_2_O_6_)

The composition of the matrix (aluminates and ferrites) is not altered by the level of SO_3_ in the clinker [6]. In particularly the amount of the tricalcium aluminate (Ca_3_Al_2_O_6_) is not affected by the SO_3_ level [2, 7]. This conclusion was not compatible with the observation of Hamou-Tagnit and Sarker, indicating that increasing the SO_3_ level increases the amount of Ca_3_Al_2_O_6_ in the clinker [8] where the conclusion of Borgholm and Jons showed the opposite [9].

These contradictions in the literature were due to the fact that many parameters could affect the development of C_3_A (Ca_3_Al_2_O_6_) principally the kiln atmosphere and the raw meal chemistry. Locher and others [10] detect that the kiln atmosphere, especially the amount of oxygen, had an influence on the amount of C_3_A. Reducing kiln atmosphere inhibits partly the oxidation of the bivalent iron (Fe^2+^), presented in the kiln feed, leading to increase in the amount of C_3_A. 

The sulphur introduced into the cement kiln from both sides, the kiln feed and kiln burner, is in chemically reduced form such as S^0^, S^1−^, and S^2−^. These sulphur forms are oxidized to S^4+^ and S^6+^ in the kiln system. This oxidizing process consumes some oxygen quantity leading to a reduction in the amount of oxygen in the kiln system. 

The presence of sodium oxide in the kiln feed affects the amount of C_3_A [11]. One of the forms of sodium oxide in the clinker is the Na_2_O · 8CaO · 3Al_2_O_3_· At clinkering temperature this compound reacts with sulphur to produce the Na_2_SO_4_ and C_3_A [11]. 

This study will focus only on the influence of the SO_3_ on the development of C_3_A (Ca_3_Al_2_O_6_). The other factors listed above are controlled to avoid any interaction with the results.

## 4. Experimental Procedure

The development of the Tricalcium aluminates Ca_3_Al_2_O_6_, in the cement kiln production, is affected by many parameters such as the kiln atmosphere [10] and the amount of sodium oxide in the raw feed [11]. This could be the reason of the large contradiction in the conclusion of the reported investigations.

In order to avoid any interactions, not only from the chemical compounds but also that related to the kiln operation, the clinker samples were selected in a stable kiln production operation conditions. All samples are commercial clinker, sampled from Cimenterie Nationale SAL. The free lime of the clinker was less than 1% and the percentage of the kiln inlet oxygen was around 3%. The samples were analyzed immediately, to avoid any influence from storage and humidity.

The variation of the clinker SO_3_ was made by changing the fuel type in the main burner as follows:

1. fuel oil containing 2.0% S,
2. petroleum coke containing 4.5% S,
3. petroleum coke containing 6.0% S.

The chemical and mineralogical analyses were done respectively according to ASTM C114 and ASTM C1365 standards. The calibration of the ARL 9800 was done using NIST standards. The fusion Claisse machine and the Herzog HTP 40 press were used for the sample preparation of the chemical and mineralogical analysis. The KOSH (potassium hydroxide and sucrose) method was implemented in order to detect the percentage of SO_3_ and Al_2_O_3_ in the silicate phases.

## 5. Results and Discussion

**Figure 1:**
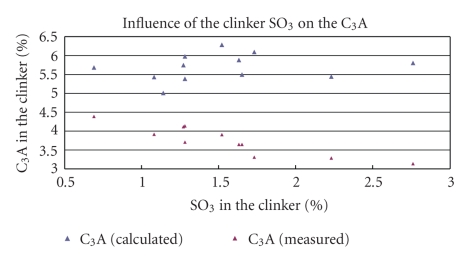
Influence of the clinker SO_3_ on the C_3_A.

The results (Figure 1) showed an obvious influence of the SO_3 _percentages on the amount of C_3_A in the low alkali clinker. In all samples, the measured percentages of C_3_A were lower than the calculated.

The correlation between the amount of C_3_A (Ca_3_Al_2_O_6_) and the total percentages of clinker SO_3_ was linear till the clinker SO_3_ reached just 2%. Over that the influence of the clinker SO_3_ becomes indistinguishable since the standard deviation of the results according to ASTM C1365:06 is 0.47.

The results in Table 1 show that the ratio of the aluminium oxide and SO_3 _in the silicate phases vary from 4.18 in the low clinker sulphur, to 1.33 in the high one.

Bonafous and other [12] noticed that this ratio is 2.  This conclusion was based on their finding that in the presence of sulphur, 3Si^4+^, in the silicate phases, is substituted by 2Al^3+^ + S^6+^.

Taylor [13] declared that this ratio is nearly more than 2, even in individual X-ray microanalyses, due to the presence of other substitutions and the accuracy of the results.

Our findings and especially the results of the samples 2 to 10 conform to the previous conclusions. 

The reason of the high ratio in the low sulphur clinker is coming from the fact that at low temperature the Al^3+^ incorporates first in the silicate phases. 

The alumina incorporated in the silicate phases is divided into two groups. The first one enters the structure at low temperature and without the influence of sulphur. In the absence of SO_3_ there is an excess of nontetrahedral cations and the number of oxygen atoms lie close to the ideal number for stoichiometric Ca_2_SiO_4 _[14]. This suggests interstitial stuffing of large cation as the main mechanism for accommodating Al on the Si site before significant solid solution of S takes place [14]. The second one is incorporated in the silicate phases with the influence of sulphur. This phenomenon is improved when the temperature exceed the 1200°C [12].

In the first sample, the ratio Al^3+^/S^6+^ is 4.18. In this case, part of the alumina is incorporated into the silicate phases without the influence of sulfate.

The amount of SO_3_ in the silicate phases is 0.22% and that for Al_2_O_3_ is 0.92 (Table 1). 

Based on the findings of Bonafous and other [12] the ratio Al^3+^/S^6+^ is 2. The calculated amount of alumina entered into the silicate phase crystals with the influence of sulphur in the first sample is 0.22 × 2 = 0.44%. The amount of alumina entered into the silicate phase crystals without the influence of sulphur in the first sample is 0.92 – 0.44 = 0.48%. 

The entry of alumina into silicate phase reduces the amount available for the C_3_A formation. The amount, of the first group, is influenced to an important degree by the changes in the composition of the ferrite compound [15] and it is compensated by the replacement of Al^3+^ by other ions in the C_3_A crystal, mainly the Si^4+^ and Fe^3+^ [16]. These phenomena lead to minimize the effect of the first group on the amount of C_3_A. The measured amount of the C_3_A becomes less than that calculated by Bogue by an average of only 3% [15]. 

The amount of sulphur in the silicate phases depend on the percentages of belite. The concentration of sulfate in belite is 4 to 5 time that in alite [13]. Regarding the second group, the incorporation of sulfate in the Alite and Belite, tends to increase the amount of alumina in the silicate phases [16]. This phenomenon was shown in the clinker samples 2 to 10. The incorporation of alumina was increased, in correlation with the sulfate, in the silicate phases. The ratio of Al_2_O_3_/SO_3_, in the silicate phase, became around 2. 

Bonafous and other [12] explain the ability of silicate phases, principally the belite, to accept simultaneously Al and S at higher dosage by the existence of synergism between both. The presence of AlO_4_^5−^decreases the negative charge induced by the substitution of SO_4_^2−^ for SiO_4_^4−^ [12]. The calculation of the sulphate phases becomes inaccurate when the percentage of clinker SO_3_ exceeds 1%. The reason for that is related to the SO_3_/Alkali ratio. At lower ratio, sulphate preferably combines with alkali to generate the Acranite K_2_SO_4_ and Aphtihitalite (K_3_Na (SO_4_)_2_) [17]. Increasing the ratio leads to the development of calcium langbeinite (Ca_2_K_2_ (SO_4_)_3_) [17]. In remarkably high ratios of SO_3_/Alkali and SO_3_ content, the anhydrite CaSO_4_ has been detected in particular clinkers [14, 18, 19].

The incorporation of alumina in the silicate phases seams to stop (samples 11 and 12) when the clinker SO_3_ exceeds the 2%.

Our result conforms to previous findings. Taylor calculated the maximum probable amount of SO_3_ in the silicate phases to be about 0.8% [13]. Miller and Tang found the largest amount of SO_3_ present in the silicate phases to be 0.68% [20]. The extra amount of sulphate shown in the over 2% clinker SO_3_ (samples 11 and 12) could come from the presence of anhydrite in the solid phase since it is not in correlation with the alumina.

The first group of alumina was calculated from the first sample with the lower clinker sulphate. The calculation of the second group of alumina in the silicate phase was done by detecting the amount of the first group (0.48) from the total amount of alumina in the silicate phases. 

**Figure 2:**
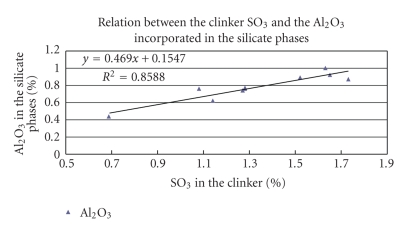
Relation between the total clinker SO_3_ and the alumina incorporated in the silicate phases forced by sulphur.

The correlation between the total clinker SO_3_ and the amount of alumina incorporated in the silicate phases, under the influence of sulphur, is acceptable (Figure 2). The percentages of alumina in the silicate phase, forced by the sulphur, became 0.469 × %SO_3_ + 0.15.

The first group of alumina incorporated in the silicate phases, without the sulphur influence, is compensated by the substitution of other elements, where as the second one is not (Figure 1).

The impurities are one of the main factors of the stabilization of various clinker crystalline forms. The most important consequence of the occurrence of impurities in the lattices of matrix clinker compounds is a disagreement between the calculated and real amount of phases in clinker. 

The amount of C_3_A in high sulphur clinker could be calculated by the following formula:

% of C_3_A = 2.65 × (%Al_2_O_3_ – (%Al_2_O_3__(Silicate  phases)_) −1.692 × %Fe_2_O_3_

(1) $$\;\;\begin{array}{cc} {\text{C}}_{3}\text{A} & =\;2.65\;\times \;\left(\%{\text{Al}}_{2}{\text{O}}_{3}-\left(0.469\;\times \;\%{\text{SO}}_{3}\;+\;0.15\right)\right)\\ & \;-1.692\;\times \;\%{\text{Fe}}_{2}{\text{O}}_{3}. \end{array}$$

In the proposed formula, the percentage of SO_3 _will be equal to 2 when the clinker SO_3_ exceeds the 2%, because over this limit the amount of alumina, in the silicate phases, becomes constant regardless the clinker SO_3_.

The calculated C_3_A amount by the new formula is more realistic than that calculated by Bogue formula (Table 2).

## 6. Conclusion

Increasing the amount of SO_3_ in the low alkali clinker decreases the percentages of C_3_A due to the high incorporation of alumina in the silicate phases. The correlation is linear till the clinker SO_3_ reaches the 2%. Over that the SO_3_ influence became undetectable.

In order to be more realistic, the proposed calculation of the C_3_A percentages takes into consideration the Al_2_O_3_ loss. The outcome shows that the new calculated results match more closely with the measured one, than with those calculated by Bogue formula.

## Figures and Tables

**Table 1:** Chemical and mineralogical analysis results.

	Al_2_O_3_	Fe_2_O_3_	SO_3_	Na_2_O	K_2_O	C_3_A (calculated)	C_3_A (measured)	SO_3_(Silicate phases)	Al_2_O_3_(Silicate phases)	Al_2_O_3_/ SO_3_
1	4.62	3.88	0.69	0.10	0.37	5.68	4.38	0.22	0.92	4.18
2	4.53	3.89	1.08	0.10	0.26	5.42	3.91	0.55	1.24	2.25
3	4.48	4.06	1.14	0.10	0.28	5.00	2.72	0.51	1.10	2.16
4	4.63	3.86	1.27	0.10	0.26	5.74	4.11	0.60	1.22	2.03
5	4.45	3.79	1.28	0.10	0.33	5.38	3.70	0.56	1.25	2.23
6	4.75	3.91	1.28	0.09	0.21	5.97	4.13	0.67	1.24	1.85
7	4.84	3.87	1.52	0.09	0.27	6.28	3.90	0.68	1.37	2.01
8	4.74	3.95	1.63	0.10	0.31	5.88	3.64	0.71	1.48	2.08
9	4.51	3.82	1.65	0.09	0.34	5.49	3.64	0.66	1.40	2.12
10	4.73	3.81	1.73	0.09	0.35	6.09	3.30	0.65	1.35	2.08
11	4.51	3.85	2.23	0.10	0.39	5.44	3.28	0.80	1.29	1.61
12	4.55	3.70	2.76	0.10	0.33	5.80	3.13	1.05	1.40	1.33

**Table 2:** Comparison of the C_3_A results.

C_3_A calculated by Bogue formula	C_3_A calculated by the new formula	C_3_A measured according to ASTMC1365:06
5.68	4.42	4.38
6.09	3.54	3.30
6.28	3.99	3.90
5.44	2.55	3.28
5.00	3.19	2.72
5.38	3.39	3.70
5.80	2.91	3.13
5.42	3.68	3.91
5.88	3.45	3.64
5.49	3.04	3.64
5.74	3.76	4.11
5.97	3.98	4.13
